# Platelet-lymphocyte ratio predicts chemotherapy response and prognosis in patients with gastric cancer undergoing radical resection

**DOI:** 10.3389/fonc.2024.1279011

**Published:** 2024-03-06

**Authors:** Qingnuo Zeng, Shilong Wang, Zilong Bai, Yuanhua Nie, Longwen Xu, Dongmin Chang

**Affiliations:** Department of Oncology Surgery, First Affiliated Hospital of Xi’an Jiaotong University, Xi’an, China

**Keywords:** gastric cancer, inflammation, platelet-lymphocyte ratio, adjuvant chemotherapy, prognosis

## Abstract

**Background:**

Amounting literatures have reported the significance of systemic inflammatory markers for evaluating tumor prognosis. But few studies have systematically compared their superiority and their impact on adjuvant chemotherapy.

**Aims:**

We aimed to investigate the ability of inflammatory markers to predict the efficacy of chemotherapy in GC patients undergoing radical therapy and to identify an effective methodology based on the study’s findings that would enable clinicians to differentiate between chemotherapy-responsive populations.

**Methods:**

We retrospectively enrolled 730 GC patients who underwent radical gastrectomy. Fibrinogen (FIB), platelet-lymphocyte ratio (PLR), systemic inflammation response index (SIRI), prognostic nutritional index (PNI), systemic immune-inflammation index (SII), neutrophil-lymphocyte ratio (NLR) and lymph node ratio (LNR) were grouped according to cutoff values. Their clinical significance for GC prognosis was determined by multivariate COX regression analysis in the 730 GC patients and high/low PLR status subgroups. Cases were divided into four groups according to PLR status and adjuvant chemotherapy status and survival was compared among groups.

**Results:**

Multivariate analysis showed that PLR was an independent prognostic factor for overall survival (OS) and disease-free survival (DFS) of GC patients. Adjuvant chemotherapy improved survival more significantly in patients with low PLR than that with high PLR. Among patients receiving adjuvant chemotherapy, low PLR was significantly associated with prolonged survival in TNM stage II, but not in TNM stage III.

**Conclusion:**

Preoperative high PLR is an independent risk factor for GC patients undergoing radical gastrectomy and adversely affects the postoperative chemotherapy effect.

## 1 Introduction

Gastric cancer (GC) is the fifth most commonly diagnosed and the fourth leading causes of cancer-related mortality worldwide (1). More than 70% of patients are diagnosed with advanced GC, which seriously threatens human health (2). Despite advancements in surgical techniques and other treatment methods, patients with advanced gastric cancer have a poor prognosis, with a median overall survival (OS) of only 1 year (3). In order to improve the OS of GC patients, it is of great clinical significance to explore the reliable prognostic markers that can help identify high-risk patients early, and take individualized treatment.

Currently, general treatment of cancer includes neoadjuvant chemotherapy and adjuvant chemotherapy, administered before and after surgery, respectively (4). However, chemoresistance remains the uppermost disincentive for cancer treatment. Chemoresistance usually display resistance through various mechanisms including tumor cell intrinsic factors and non-tumor cell extrinsic factors. The latter includes the tumor-associated inflammatory microenvironment (5). Notably, indexes of inflammatory cells, including the pretreatment neutrophil-lymphocyte ratio (NLR) lymphocyte-monocyte ratio (LMR) and platelet-lymphocyte ratio (PLR), can reflect the extent of inflammation. Studies have suggested that the combination C-reactive protein (CRP), systemic inflammation response index (SIRI) and PLR not only predicts OS, relapse-free survival (RFS), but also significantly correlates with the degree of lymph node metastasis in GC (6). Inflammatory are closely related not only to cancer prognosis (7–9), but also to chemotherapy response (10). However, the evidence is still limited regarding the joint association between high inflammation condition and the prognosis in patients with GC. Controversy remains about the effect of inflammation on the response to chemotherapy in patients with GC.

In this study, we aimed to investigate the ability of inflammatory markers to predict the efficacy of chemotherapy in patients with GC treated with curative treatment and to identify an effective method that would allow clinicians to differentiate between chemotherapy-responsive populations.

## 2 Materials and methods

### 2.1 Patients

We retrospectively enrolled 730 patients with primary GC who underwent radical resection between January 2010 and December 2017 at The First Affiliated Hospital of Xi’an Jiaotong University, Xi’an, China. All patients were diagnosed based on pathological evidence and staged according to the eighth edition of American Joint Committee on Cancer (AJCC) tumor-node-metastasis (TNM) staging system. Patient follow-up data were obtained through regular follow-up with a final follow-up time of June 2020. OS was defined as the time from the date of radical surgery to the time of last follow-up or time of death, and disease-free survival (DFS) was defined as the time from the date of radical surgery to the time of last follow-up or time of recurrence. For OS, the endpoint event was death. For DFS, the endpoint event was disease recurrence, and censoring meant that no endpoint event was observed at the last follow-up. Recurrence is diagnosed based on imaging findings or biopsy of suspicious lesions. Adjuvant chemotherapy is recommended for most pathological stage II and III patients in our center according to the patient’s wishes and health status. Our center routinely recommends a combination of 5-fluorouracil and cisplatin/oxaliplatin or paclitaxel chemotherapy regimens. Inclusion criteria: (1) all patients were initially diagnosed and had pathological evidence; (2) stage I-III disease; (3) age≥18 years; (4) patients with pathologically negative resection margins (R0 resection); (5) complete clinical data. Exclusion criteria: (1) accompanying or secondary to other tumors; (2) infection, inflammation, hematologic disease or taking medications that affect hematology 1 months before surgery; (3) received any treatment prior to radical gastrectomy; (4) lost to follow-up. Laboratory indicators were within one week before treatment. All methods were performed in accordance with the relevant guidelines and regulations. Informed consent was obtained from all subjects and/or their legal guardian(s).

### 2.2 Baseline characteristics and optimal cut-off values

We collected gender, age at surgery, hematologic data (including complete blood count, fibrinogen (FIB) and albumin), pathological parameters (tumor location, tumor differentiation, T stage, N stage, TNM stage, lymph nodes retrieval and tumor size) and adjuvant chemotherapy status. PLR = platelet count/lymphocyte count. SIRI = neutrophil count * monocyte count/lymphocyte count (11). NLR = neutrophil count/lymphocyte count. Prognostic nutritional index (PNI) = (10 * serum albumin, g/dl) + (0.005 * blood lymphocyte count, unit/l). Systemic immune-inflammation index (SII) = platelet count * neutrophil count/lymphocyte count. Lymph node ratio (LNR) was calculated by dividing the number of tumor cell positive lymph nodes by the number of resected lymph nodes. The cut-off value for age was set to 60 years and the cut-off value for tumor size was 5 cm. The cut-off values of other parameters were calculated by receiver operating characteristics (ROC) curve analysis. The evaluation criterion of ROC analysis was whether the patient died at the last follow-up. Tumor histology was divided into undifferentiated type (including undifferentiated or poorly differentiated adenocarcinoma, mucinous carcinoma and signet ring cell carcinoma) and differentiated type (including well or moderately differentiated adenocarcinoma).

### 2.3 Statistical analysis

Continuous variates were grouped according to their respective cut-off values and presented as frequencies and percentages and compared using the chi-square test or Fisher exact test. Continuous non-normally distributed variables variates were presented as the median and interquartile range (IQR) in parentheses and compared with log-rank tests, while continuous normally distributed variates were presented as mean ± standard deviation and compared using Student’s *t-tests*. Factors related to OS and DFS were assessed by the log-rank test and visualized using the Kaplan-Meier method. Survival rate was obtained from survival analysis table. Independent prognostic factors for OS and DFS were determined by multivariate Cox regression analysis and assessed by Wald’s test. The statistically significant variables from the univariate analysis were included in multivariate analysis. Inflammatory markers that are independent risk factors for GC are used for subsequent subgroup analysis. To explore the effect of inflammatory status on adjuvant chemotherapy, univariate and multivariate Cox regression analysis was also conducted in cohort stratified by inflammation status.

Statistical analysis and plotting were performed with SPSS Statistics software (version 22.0, IL, USA), 2-sided *p*<0.05 were considered statistical significantly.

## 3 Result

### 3.1 Clinical characteristics of the patients

A total of 730 GC patients were included. There were 178 (24.4%) female and 398 (54.5%) patients older than 60 years. Distal gastric tumors accounted for 58.8% of all tumors, and proximal tumors accounted for 24.5%. In terms of GC staging, there were 199, 101, and 430 patients with GC stages I, II, and III, respectively. Among all samples, there were 512 (70.1%) cases undifferentiated and 238 (32.6%) cases with tumor size greater than or equal to 5 cm. 445 (61%) patients received adjuvant chemotherapy (**Table 1**).

**Table 1:** Association between baseline characteristics and PLR (N=730).

Characteristics	All	Low PLR	High PLR	P value
	(N=730)	(N=509)	(N=221)	
Gender, female	178(24.4)	106(20.8)	72(32.6)	0.001
Age, ≥60 years	398(54.5)	276(54.2)	122(55.2)	0.807
FIB, ≥3.585 g/l	245(33.6)	139(27.3)	106(48)	<0.001
SIRI, ≥0.665	403(55.2)	249(48.9)	154(69.7)	<0.001
PNI, ≥40.06	460(63)	312(61.3)	148(67)	0.145
SII, ≥456.3	298(40.8)	120(23.6)	178(80.5)	<0.001
NLR, ≥2.08	322(44.1)	160(31.4)	162(73.3)	<0.001
LNR, ≥0.085	292(40)	201(39.5)	91(41.2)	0.387
Tumor size, ≥5 cm	238(32.6)	138(27.1)	100(45.2)	<0.001
Histology				0.218
differentiated	218(29.9)	159(31.2)	59(26.7)	
undifferentiated	512(70.1)	350(68.8)	162(73.3)	
Tumor location				0.692
proximal stomach	179(24.5)	123(24.2)	56(25.3)	
distal stomach	429(58.8)	297(58.3)	132(59.7)	
total stomach	122(16.7)	89(17.5)	33(14.9)	
T stage				<0.001
T1	170(23.3)	138(27.1)	32(14.5)	
T2	61(8.4)	49(9.6)	12(5.4)	
T3	77(10.5)	53(10.4)	24(10.9)	
T4	422(57.8)	269(52.8)	153(69.2)	
N stage				0.38
N0	327(44.8)	237(46.6)	90(40.7)	
N1	116(15.9)	82(16.1)	34(15.4)	
N2	123(16.8)	83(16.3)	40(18.1)	
N3	164(22.5)	107(21)	57(25.8)	
TNM stage				<0.001
I	199(27.3)	162(31.8)	37(16.7)	
II	101(13.8)	69(13.6)	32(14.5)	
III	430(58.9)	278(54.6)	152(68.8)	
Chemotherapy				0.043
yes	445(61)	298(58.5)	147(66.5)	
no	285(39)	211(41.5)	74(33.5)	
OS, month	41(27-63.25)	41(31-65)	40(19-58.5)	0.019
DFS, month	41(25-63)	41(28-65)	40(19-58)	0.025

Data are presented as quantity and percentage or median and interquartile range in parentheses. FIB, fibrinogen; PLR, platelet lymphocyte ratio; SIRI, systemic inflammation response index; PNI, prognostic nutritional index; SII, systemic immune-inflammation index; NLR, neutrophil-lymphocyte ratio; LNR, lymph node ratio. TNM, tumor node metastasis; OS, overall survival; DFS, disease-free survival. Chemotherapy, refers to adjuvant chemotherapy. The cut-off value of PLR was 163.8 obtained from ROC curve.

### 3.2 The value of FIB, PLR, SIRI, PNI, SII, NLR in the prognosis prediction of gastric cancer

**Figure 1:**
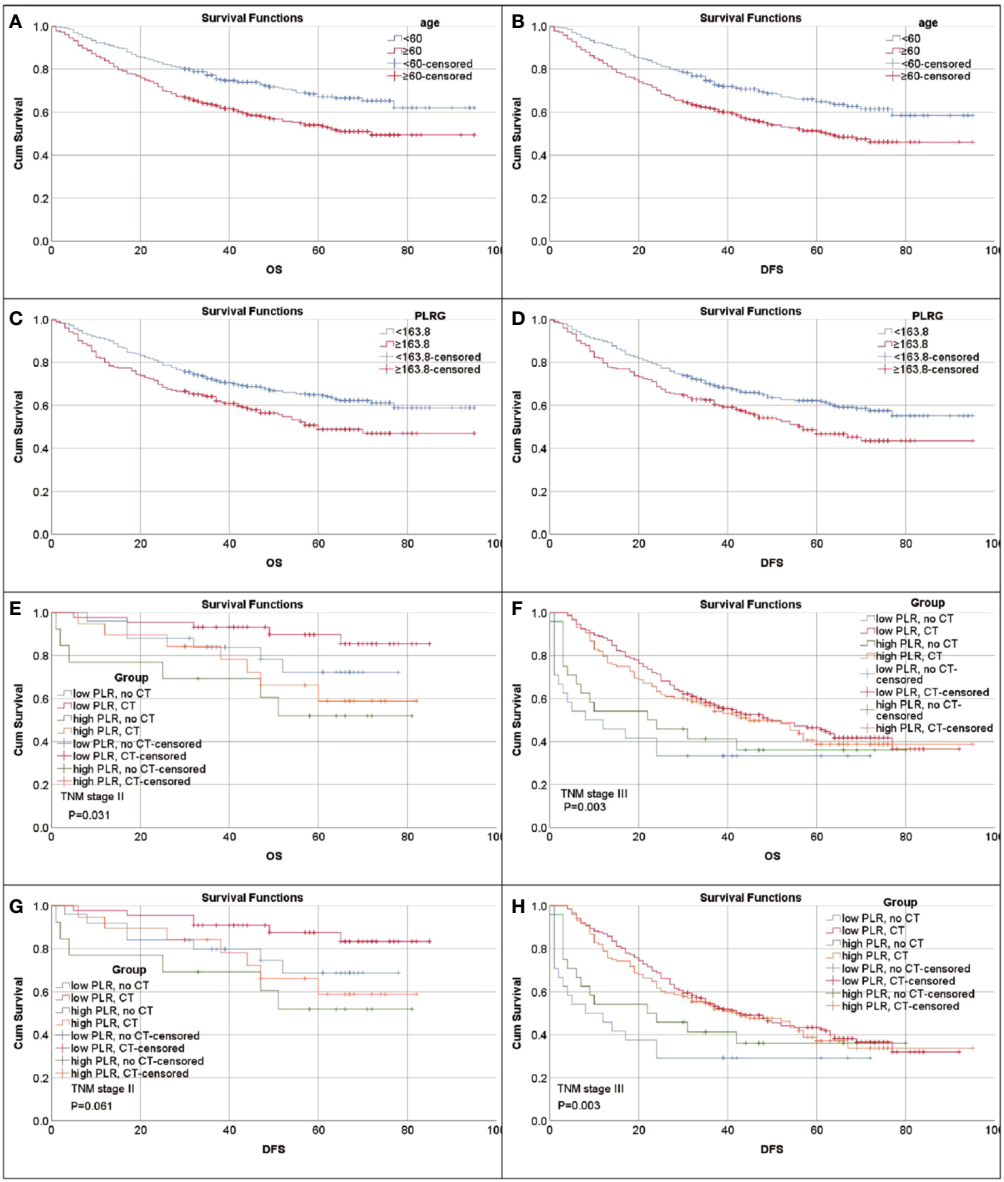
Kaplan-Meier survival curves for patients with gastric cancer. **(A)** Survival curves of age for OS. **(B)** Survival curves of age for DFS. **(C)** Survival curves of PLR for OS. **(D)** Survival curves of PLR for DFS. **(E–H)** PLR can identify patient response to adjuvant chemotherapy (CT). **(E)** Survival curves of PLR-CT groups for OS in patients with stage II. Low PLR, no CT vs. CT P=0.158; high PLR, no CT vs. CT P=0.524. **(F)** Survival curves of PLR-CT groups for OS in patients with stage III. Low PLR, no CT vs. CT P=0.001; high PLR, no CT vs. CT P<0.107. **(G)** Survival curves of PLR-CT groups for DFS in patients with stage II. Low PLR, no CT vs. CT P=0.125; high PLR, no CT vs. CT P<0.524. **(H)** Survival curves of PLR-CT groups for DFS in patients with stage III. Low PLR, no CT vs. CT P<0.001; high PLR, no CT vs. CT P=0.174.

The optimal cut-off values for FIB, PLR, SIRI, PNI, SII, NLR and LNR were 3.585 g/l, 163.8, 0.665, 40.06, 456.3, 2.08 and 0.085, respectively (**Supplementary Figure 1**). Kaplan-Meier survival analysis showed that older GC patients had worse OS and DFS than younger patients (**Figures 1A, B**), and patients with higher PLR had worse OS and DFS than GC patients with lower PLR (**Figures 1C, D**). High FIB, high SIRI, low PNI, high SII and high NLR were also associated with poor OS (**Supplementary Figure 2**). Consistently, in univariate analysis, we found that age, FIB, PLR, SIRI, PNI, SII, NLR, LNR, tumor size, tumor location, tumor differentiation, TNM stage and adjuvant chemotherapy were associated with OS. In the multivariate analysis, higher PLR was an independent risk factor for GC with a hazard ratio (HR) of 1.413 and a 95% confidence interval (CI) of 1.069-1.866 (P=0.015). Likewise, elder age (HR: 1.424, 95% CI: 1.082-1.873) (P=0.012), higher FIB (HR: 1.322, 95% CI: 1.008-1.734) (P=0.044), higher LNR (HR: 2.77, 95% CI: 1.95-3.934) (P<0.001) and advanced TNM stage with a HR (95% CI) of 3.002 (1.477-6.101) for stage II (P=0.002) and 6.125 (3.125-12.005) for stage III (P<0.001) were also independent risk factors. While adjuvant chemotherapy was a protective factor (HR: 0.476, 95% CI: 0.33-0.686) (P<0.001) (**Table 2**). For DFS, higher PLR (HR: 1.396, 95% CI: 1.072-1.818) (P=0.013), elder age (P=0.005), higher LNR (P<0.001), without adjuvant chemotherapy (P<0.001) and advanced stage (P=0.002 for stage II and P<0.001 for stage III) as risk factors also showed significance. To our surprise, FIB was not an independent risk factor for DFS (**Table 3**). When we focused on patients who received adjuvant chemotherapy and performed subgroup analyses according to TNM stage, we found that low PLR was significantly associated with prolonged survival (both OS and DFS) in TNM stage II (P=0.024 for OS, P=0.043 for DFS), but not in TNM stage III (P=0.418 for OS, P=0.548 for DFS) (**Supplementary Figures 3A–D**).

**Table 2:** Univariate and multivariate analyses for overall survival of GC patients (N=730).

Parameters	Univariate analysis			Multivariate analysis		
	HR	95%CI	P value	HR	95%CI	P value
Gender	1.147	0.865-1.52	0.34			
Age	1.678	1.311-2.148	<0.001	1.424	1.082-1.873	0.012
FIB	1.752	1.381-2.224	<0.001	1.322	1.008-1.734	0.044
PLR	1.513	1.184-1.933	0.001	1.413	1.069-1.866	0.015
SIRI	1.575	1.23-2.018	<0.001			0.176
PNI	0.489	0.371-0.644	<0.001			0.121
SII	1.462	1.153-1.853	0.002			0.959
NLR	1.466	1.157-1.859	0.002			0.451
LNR	4.534	3.393-6.059	<0.001	2.77	1.95-3.934	<0.001
Tumor size	1.96	1.51-2.543	<0.001			0.154
Tumor location						
proximal stomach	1					
distal stomach	0.695	0.524-0.921	0.011			0.482
full stomach	1.488	1.061-2.085	0.021			0.065
Histology	0.84	0.646-1.092	0.192			
TNM stage						
I	1			1		
II	2.387	1.319-4.321	0.004	3.002	1.477-6.101	0.002
III	7.404	4.684-11.703	<0.001	6.125	3.125-12.005	<0.001
Chemotherapy^1^	2.439	1.84-3.234	<0.001	0.476	0.33-0.686	<0.001

HR, hazard ratio; CI, confidence interval. FIB, fibrinogen; PLR, platelet-lymphocyte ratio; SIRI, systemic inflammation response index; PNI, prognostic nutritional index; SII, systemic immune-inflammation index; NLR, neutrophil-lymphocyte ratio; LNR, lymph node ratio; TNM, tumor node metastasis. The reference of parameters was female, age<60 years, FIB<3.585, PLR<163.8, SIRI<0.665, PNI<40.06, SII<456.3, NLR<2.08, LNR<0.085, tumor size<5 cm, undifferentiated and without adjuvant chemotherapy, respectively. ^1^Adjuvant chemotherapy appeared to be a risk factor in univariate analysis because patients with TNM stage I were included in COX regression. In fact, in the univariate analysis considering only TNM stage II-III, the HR for adjuvant chemotherapy was 0.222 (95% CI: 0.168-0.292, P<0.001).

**Table 3:** Univariate and multivariate analyses for disease-free survival of GC patients (N=730).

Parameters	Univariate analysis			Multivariate analysis		
	HR	95%CI	P value	HR	95%CI	P value
Gender	1.177	0.896-1.545	0.241			
Age	1.624	1.283-2.056	<0.001	1.449	1.12-1.876	0.005
FIB	1.723	1.369-2.167	<0.001			0.06
PLR	1.461	1.153-1.851	0.002	1.396	1.072-1.818	0.013
SIRI	1.503	1.186-1.905	0.001			0.185
PNI	0.514	0.396-0.668	<0.001			0.198
SII	1.499	1.193-1.883	0.001			0.525
NLR	1.478	1.177-1.857	0.001			0.197
LNR	4.358	3.314-5.73	<0.001	2.696	1.931-3.763	<0.001
Tumor size	1.901	1.481-2.439	<0.001			0.112
Tumor location						
proximal stomach	1					
distal stomach	0.689	0.526-0.903	0.007			0.362
full stomach	1.447	1.043-2.005	0.027			0.069
Histology	0.855	0.665-1.099	0.221			
TNM stage						
I	1			1		
II	2.159	1.24-3.761	0.007	2.752	1.431-5.292	0.002
III	6.728	4.42-10.24	<0.001	5.655	3.047-10.497	<0.001
Chemotherapy^1^	2.42	1.848-3.171	<0.001	0.499	0.351-0.711	<0.001

The reference of parameters was female, age<60 years, FIB<3.585, PLR<163.8, SIRI<0.665, PNI<40.06, SII<456.3, NLR<2.08, LNR<0.085, tumor size<5 cm, undifferentiated and without adjuvant chemotherapy, respectively. ^1^In the univariate analysis considering only TNM stage II-III, the HR for adjuvant chemotherapy was 0.238 (95% CI: 0.181-0.312, P<0.001).

### 3.3 PLR adversely affects adjuvant chemotherapy efficacy

To explore the impact of inflammatory status on tumor treatment and prognosis, we performed subgroup analysis based on PLR levels. Baseline Characteristics showed that a high PLR was associated with gender (P=0.001), FIB (P<0.001), SIRI (P<0.001), SII (P<0.001), NLR (P<0.001), tumor size (P<0.001), TNM stage (P<0.001) and chemotherapy (P=0.043) (**Table 1**). As previously described, variables that were statistically significant in univariate analysis were included in multivariate Cox regression analysis. We included adjuvant chemotherapy in the multivariate analysis regardless of whether there was a statistical difference in the univariate analysis. Multivariate analysis showed that elevated FIB (HR: 1.592, 95% CI: 1.13-2.244, P=0.008), higher LNR (HR: 2.189, 95% CI: 1.388-3.45, P=0.001), advanced TNM stage (stage II, HR: 2.624, 95% CI:1.014-6.789, P=0.047; stage III, HR: 10.398, 95% CI: 4.287-25.223, P<0.001) were found to be risk factors in patients with low PLR. As expected, adjuvant chemotherapy was associated with favorable prognosis (HR: 0.402, 95% CI: 0.238-0.677, P=0.001) (**Table 4**). In the high PLR subgroup, to our surprise, adjuvant chemotherapy is not associated with GC survival in univariate survival analysis (P=0.055). Multivariate survival analysis showed that elder age (HR:1.609, 95% CI: 1.036-2.499, P=0.034), higher SIRI (HR: 1.965, 95% CI: 1.104-3.499, P=0.022) and higher LNR (HR: 3.53, 95% CI: 2.22-5.613, P<0.001) was significantly associated with shorter OS (**Table 5**). While chemotherapy was not associated with OS (P=0.055). In terms of DFS, after subgrouping by PLR, univariate survival analysis and multivariate survival analysis yielded similar results to OS (**Supplementary Tables 1**, **2**). To explore whether the effect of inflammation on chemotherapy efficacy is stage-specific, cases were divided into four groups according to PLR status and adjuvant chemotherapy. The Kaplan-Meier curves were used to determine OS and DFS of GC patients with stage II or stage III. In TNM stage II, the effect of adjuvant chemotherapy in the low PLR group was significantly higher than that in the high PLR group (**Figures 1E, G**). Although survival was comparable between the low and high PLR groups among patients with TNM stage III receiving chemotherapy, adjuvant chemotherapy improved survival more significantly in the low PLR group than in the high PLR group, which is consistent with the cox multivariate analysis results (**Figures 1F, H**).

**Table 4:** Univariate and multivariate analyses for overall survival of GC patients with low PLR (N=509).

Parameters	Univariate analysis			Multivariate analysis		
	HR	95%CI	P value	HR	95%CI	P value
Gender	1.189	0.81-1.746	0.378			
Age	1.681	1.231-2.295	0.001			
FIB	2.141	1.581-2.899	<0.001	1.592	1.13-2.244	0.008
SIRI	1.381	1.022-1.867	0.036			
PNI	0.523	0.373-0.733	<0.001			
SII	1.314	0.937-1.843	0.113			
NLR	1.429	1.047-1.949	0.024			
LNR	5.015	3.452-7.285	<0.001	2.189	1.388-3.45	0.001
Tumor size	2.179	1.56-3.043	<0.001			
Tumor location						
proximal stomach	1					
distal stomach	0.624	0.438-0.89	0.009			
full stomach	1.454	0.961-2.201	0.076			
Histology	0.894	0.647-1.237	0.499			
TNM stage						
I	1			1		
II	1.692	0.777-3.683	0.185	2.624	1.014-6.789	0.047
III	7.848	4.608-13.369	<0.001	10.398	4.287-25.223	<0.001
Chemotherapy^1^	2.992	2.078-4.307	<0.001	0.402	0.238-0.677	0.001

The reference of parameters was female, age<60 years, FIB<3.585, PLR<163.8, SIRI<0.665, PNI<40.06, SII<456.3, NLR<2.08, LNR<0.085, tumor size<5 cm, undifferentiated and without adjuvant chemotherapy, respectively. ^1^In the univariate analysis considering only TNM stage II-III, the HR for adjuvant chemotherapy was 0.149 (95% CI: 0.104-0.213, P<0.001).

**Table 5:** Univariate and multivariate analyses for overall survival of GC patients with high PLR (N=221).

Parameters	Univariate analysis			Multivariate analysis		
	HR	95%CI	P value	HR	95%CI	P value
Gender	1.277	0.837-1.948	0.257			
Age	1.673	1.117-2.505	0.013	1.609	1.036-2.499	0.034
FIB	1.083	0.734-1.597	0.688			
SIRI	1.756	1.095-2.815	0.019	1.965	1.104-3.499	0.022
PNI	0.449	0.278-0.727	0.001			0.092
SII	1.15	0.691-1.913	0.592			
NLR	1.095	0.705-1.7	0.687			
LNR	3.8	2.393-6.034	<0.001	3.53	2.22-5.613	<0.001
Tumor size	1.317	0.859-2.018	0.206			
Tumor location						
proximal stomach	1					
distal stomach	0.839	0.526-1.338	0.461			
full stomach	1.63	0.909-2.922	0.101			
Histology	0.791	0.504-1.243	0.309			
TNM stage						
I	1					
II	3.152	1.123-8.844	0.029			0.107
III	5.604	2.271-13.831	<0.001			0.742
Chemotherapy^1^	1.547	0.99-2.417	0.055			0.061

The reference of parameters was female, age<60 years, FIB<3.585, PLR<163.8, SIRI<0.665, PNI<40.06, SII<456.3, NLR<2.08, LNR<0.085, tumor size<5 cm, undifferentiated and without adjuvant chemotherapy, respectively. ^1^In the univariate analysis considering only TNM stage II-III, the HR for adjuvant chemotherapy was 0.375 (95% CI: 0.24-0.587, P<0.001).

## 4 Discussion

In our retrospective analysis, we systematically explored the prognostic significance of representative blood-derived inflammatory markers. We confirmed the effect of PLR as the most prominent inflammatory marker on the survival of GC patients after radical therapy. We also demonstrated the adverse impact of inflammation on adjuvant chemotherapy.

There is a mutually reinforcing relationship between tumors and systemic inflammation (12). The prognostic value of blood-borne inflammatory markers including PLR, SIRI, PNI, SII, NLR, etc. in cancer patients has been clearly articulated (13–17). The combined prognostic value of inflammatory markers has also been reported (18, 19). However, few studies have compared the superiority of these indicators in predicting tumor prognosis. One study reported that, compared with PLR, NLR has superiority in assessing prognosis of metastatic gastric cancer (17). Conversely, the superiority of PLR in blood-derived inflammatory factors in predicting prognosis has also been reported (20). Another study reported that neither SII, NLR nor PLR were independent factors for OS (21). Based on these inconsistencies, the relationship between inflammatory markers and tumor prognosis needs to be further explored. Here, we found that PLR and FIB were independent prognostic factors in patients with GC by multivariate survival analysis. patients with PLR>163.8 or FIB>3.585 had significantly worse OS and DFS. As tumor-associated inflammation can enhance neo-angiogenesis, promote tumor progression and metastatic spread, cause local immunosuppression, and further increase genomic instability (22), the clinical significance of the optimal inflammatory marker PLR is taken for granted. When we performed subgroup analysis by PLR level, we found that adjuvant chemotherapy did not significantly improve survival in patients with high PLR level. Contrastingly, adjuvant chemotherapy in the low PLR subgroup demonstrated significance in assessing survival. In other words, in a hyperinflammatory state, the effect of chemotherapy is limited. The influence of inflammatory status on the efficacy of chemotherapy was presented by Kaplan-Meier curves. As mentioned, low PLR patients receiving chemotherapy show best prognosis. In the low-PLR subgroup, the chemotherapy patients had a significantly longer OS and DFS than the non-chemotherapy patients. In the high PLR group, there was no significant difference in prognosis between chemotherapy patients and non-chemotherapy patients. Multivariate survival analysis in subgroups also confirmed these results. In conclusion, patients with low inflammatory status seem to be more suitable for adjuvant chemotherapy. Anti-inflammatory therapy combined with adjuvant chemotherapy may achieve better efficacy in patients with a hyperinflammatory state, especially patients with TNM stage II.

A study on PLR for predicting survival in gastric mucinous adenocarcinoma reported that the optimal cut-off value of PLR was set at 133 according to the ROC curve (13). In another study on metastatic gastric cancer, the best cut-off value for PLR was 201.6 (23). Whereas our current study found that the best cut-off value for PLR was 163.8. This is generally consistent with the results of previous literature. More accurate cutoffs may require studies with large sample sizes. Furthermore, we focused more on the role of adjuvant chemotherapy in different inflammatory states than on the prognostic value of PLR. Based on our results, combining anti-inflammatory therapy with adjuvant chemotherapy may prolong patient survival.

Multiple studies report the prognostic value of hyperfibrinogenemia in various tumors (24, 25). Plasma fibrinogen promotes tumor cell growth and angiogenesis by interacting with fibroblast growth factor-2 and vascular endothelial growth factor (26). Consistently, we validated the role of fibrinogen in the prognosis of gastric cancer. Since hyperfibrinogenemia reflects the c hypercoagulable state to a certain extent, and hypercoagulation may contribute to the hematogenous metastasis of tumors (27), it is difficult to judge how much fibrinogen directly promotes the tumor in the poor prognosis of gastric cancer. Furthermore, growing evidence suggests a broad interaction between coagulation and inflammation, with inflammation leading to activation of coagulation, and coagulation also significantly affecting inflammatory activity (28). The crosstalk between these mechanisms together contributes to the formation of a tumor-promoting microenvironment. This explains the underlying mechanism by which fibrinogen and inflammatory markers are linked to poor prognosis.

In recent years, the underlying mechanism by which platelets promote tumor progression has been elucidated. For example, platelets promote cell proliferation, angiogenesis, and epithelial-mesenchymal transition by secreting cytokines and chemical factors, and protect tumor cells from immune system attack by forming microthrombi on tumor cells (29). Not only that, tumor-platelet bidirectional interactions are closely related to chemoresistance (30). It has been reported that low platelet count enhanced the tumoricidal effects of chemotherapy in breast cancer (31). There is also evidence that thrombocytosis promotes tumor growth and inhibits ovarian cancer response to docetaxel. Chemotherapy combined with antiplatelet antibodies inhibited tumor growth more effectively (32). In fact, the antiplatelet agent aspirin inhibited platelet-mediated angiogenesis and tumor cell proliferation (33, 34). Low-dose aspirin reduces long-term morbidity and mortality from colon cancer (35). Consistent with this evidence, we found that high PLR was associated with poor prognosis and poor chemotherapy response in gastric cancer. Although studies have shown that low-dose aspirin does not improve survival in gastric or esophageal cancer (36), the role of inflammation in chemoresistance has been demonstrated (5). This relationship was also reflected in inflammatory markers. Association of high NLR values with chemoresistance and poor prognosis has been reported (37). This inspires us that blood inflammation indicators may be used as a reference for anti-inflammatory adjuvant therapy.

Our current study has some drawbacks, namely that it was a retrospective analysis with a relatively small sample of female cases. Although we recorded the status of postoperative adjuvant chemotherapy, we did not record the chemotherapy regimen in detail. The levels of various inflammatory cells in the blood are affected by many factors, such as chronic inflammation. There are also some inflammatory markers not included in the analysis, such as CRP and CRP-derived markers. It cannot be ignored that preoperative inflammatory markers did not necessarily correlate with the patient’s inflammatory status before chemotherapy. To assess the effect of inflammation on the efficacy of adjuvant chemotherapy, it is more persuasive to assess the patient’s inflammatory status during the peri-chemotherapy period. Helicobacter pylori (H. pylori) infection is the greatest risk factor associated with gastric cancer (20). Approximately 75% of the global gastric cancer burden and 5.5% of malignancies worldwide are attributable to H pylori-induced inflammation and injury (38). However, due to insufficient data on this test in diagnosed patients, we did not study it.

In conclusion, the present study validates the prognostic utility of PLR. Adjuvant chemotherapy significantly improves survival in patients with low PLR. Adjuvant chemotherapy combined with anti-inflammatory therapy may achieve better survival.

## Data availability statement

The raw data supporting the conclusions of this article will be made available by the authors, without undue reservation.

## Ethics statement

The studies involving humans were approved by Ethics Committee of First Affiliated Hospital of Xi’an Jiaotong University. The studies were conducted in accordance with the local legislation and institutional requirements. Written informed consent for participation was not required from the participants or the participants’ legal guardians/next of kin in accordance with the national legislation and institutional requirements.

## Author contributions

QZ: Writing – original draft. SW: Writing – review & editing. ZB: Writing – review & editing. YN: Writing – review & editing. LX: Writing – review & editing. DC: Writing – review & editing.

## References

[1] Sung H Ferlay J Siegel RL Laversanne M Soerjomataram I Jemal A. Global cancer statistics 2020: GLOBOCAN estimates of incidence and mortality worldwide for 36 cancers in 185 countries. CA Cancer J Clin. (2021) 71:209–49. doi: 10.3322/caac.21660 33538338

[2] Hunt RH Camilleri M Crowe SE El-Omar EM Fox JG Kuipers EJ. The stomach in health and disease. Gut. (2015) 64:1650–68. doi: 10.1136/gutjnl-2014-307595 PMC4835810 26342014

[3] Nakamura Y Kawazoe A Lordick F Janjigian YY Shitara K. Biomarker-targeted therapies for advanced-stage gastric and gastro-esophageal junction cancers: an emerging paradigm. Nat Rev Clin Oncol. (2021) 18:473–87. doi: 10.1038/s41571-021-00492-2 33790428

[4] Sun W Yan L. Gastric cancer: current and evolving treatment landscape. Chin J Cancer. (2016) 35:83. doi: 10.1186/s40880-016-0147-6 27581465 PMC5006607

[5] de Visser KE Jonkers J. Towards understanding the role of cancer-associated inflammation in chemoresistance. Curr Pharm Des. (2009) 15:1844–53. doi: 10.2174/138161209788453239 19519427

[6] Kang WZ Xiong JP Li Y Jin P Xie YB Xu Q. A new scoring system to predict lymph node metastasis and prognosis after surgery for gastric cancer. Front Oncol. (2022) 12:809931. doi: 10.3389/fonc.2022.809931 35198443 PMC8859260

[7] Miyamoto R Inagawa S Sano N Tadano S Adachi S Yamamoto M. The neutrophil-to-lymphocyte ratio (NLR) predicts short-term and long-term outcomes in gastric cancer patients. Eur J Surg Oncol. (2018) 44:607–12. doi: 10.1016/j.ejso.2018.02.003 29478743

[8] Ohe Y Fushida S Yamaguchi T Kinoshita J Saito H Okamoto K. Peripheral blood platelet-lymphocyte ratio is good predictor of chemosensitivity and prognosis in gastric cancer patients. Cancer Manag Res. (2020) 12:1303–11. doi: 10.2147/CMAR.S241069 PMC7039245 32110104

[9] Xu BB Xu Y Lu J Wu Y Wang JB Lin JX. Prognostic significance of combined Lymphocyte-monocyte Ratio and Tumor-associated Macrophages in Gastric Cancer Patients after Radical Resection. J Cancer. (2020) 11:5078–87. doi: 10.7150/jca.44440 PMC7378932 32742455

[10] Ho EA Piquette-Miller M. Regulation of multidrug resistance by pro inflammatory cytokines. Curr Cancer Drug Targets. (2006) 6:295–311. doi: 10.2174/156800906777441753 16848721

[11] Chen Y Jin M Shao Y Xu G. Prognostic value of the systemic inflammation response index in patients with adenocarcinoma of the esophagogastric junction A propensity score-matched analysis. Dis Markers. (2019) 2019:4659048. doi: 10.1155/2019/4659048 31781301 PMC6875417

[12] Balkwill F Mantovani A. Inflammation and cancer: back to Virchow? Lancet. (2001) 357:539–45. doi: 10.1016/S0140-6736(00)04046-0 11229684

[13] Zhu Z Gao J Liu Z Li C Xue Y. Preoperative platelet-to-lymphocyte ratio (PLR) for predicting the survival of stage I-III gastric cancer patients with a MGC component. BioMed Res Int. (2021) 2021:9678363. doi: 10.1155/2021/9678363 33997045 PMC8112911

[14] Li S Lan X Gao H Li Z Chen L Wang W. Systemic Inflammation Response Index (SIRI), cancer stem cells and survival of localized gastric adenocarcinoma after curative resection. J Cancer Res Clin Oncol. (2017) 143:2455–68. doi: 10.1007/s00432-017-2506-3 PMC11819166 28828692

[15] Xishan Z Ye Z Feiyan M Liang X Shikai W. The role of prognostic nutritional index for clinical outcomes of gastric cancer after total gastrectomy. Sci Rep. (2020) 10:17373. doi: 10.1038/s41598-020-74525-8 33060715 PMC7562903

[16] Hirahara N Tajima Y Matsubara T Fujii Y Kaji S Kawabata Y. Systemic immune-inflammation index predicts overall survival in patients with gastric cancer: a propensity score-matched analysis. J Gastrointest Surg. (2021) 25:1124–33. doi: 10.1007/s11605-020-04710-7 32607856

[17] Zhao G Liu N Wang S Guo J Song X Qi Y. Prognostic significance of the neutrophil-to-lymphocyte and platelet-to-lymphocyte ratio in patients with metastatic gastric cancer. Med (Baltimore). (2020) 99:e19405. doi: 10.1097/MD.0000000000019405 PMC7478543 32150090

[18] Hirahara T Arigami T Yanagita S Matsushita D Uchikado Y Kita Y. Combined neutrophil-lymphocyte ratio and platelet-lymphocyte ratio predicts chemotherapy response and prognosis in patients with advanced gastric cancer. BMC Cancer. (2019) 19:672. doi: 10.1186/s12885-019-5903-y 31286873 PMC6615151

[19] Ma M Weng M Chen F Hu Y Lai J Wang Y. Systemic inflammation score is a prognostic marker after curative resection in gastric cancer. ANZ J Surg. (2019) 89:377–82. doi: 10.1111/ans.15103 PMC6593849 30854753

[20] Hardbower DM Peek RM Jr Wilson KT. At the Bench: Helicobacter pylori, dysregulated host responses, DNA damage, and gastric cancer. J Leukoc Biol. (2014) 96:201–12. doi: 10.1189/jlb.4BT0214-099R PMC4101087 24868089

[21] Hirahara N Matsubara T Fujii Y Kaji S Kawabata Y Hyakudomi R. Comparison of the prognostic value of immunoinflammation-based biomarkers in patients with gastric cancer. Oncotarget. (2020) 11:2625–35. doi: 10.18632/oncotarget.v11i27 PMC7343633 32676164

[22] Grivennikov SI Greten FR Karin M. Immunity, inflammation, and cancer. Cell. (2010) 140:883–99. doi: 10.1016/j.cell.2010.01.025 PMC2866629 20303878

[23] Wang J Qu J Li Z Che X Liu J Teng Y. Pretreatment platelet-to-lymphocyte ratio is associated with the response to first-line chemotherapy and survival in patients with metastatic gastric cancer. J Clin Lab Anal. (2018) 32(1):e22185. doi: 10.1002/jcla.22185 28238215 PMC6817026

[24] Wang Y Xu W Wang Y. Prognostic role of preoperative fibrinogen to albumin ratio in breast cancer. Clin Chim Acta. (2020) 510:360–2. doi: 10.1016/j.cca.2020.07.055 32739214

[25] Zhang L Wang Z Xiao J Zhang Z Li H Wang Y. Prognostic value of fibrinogen-to-albumin ratio in patients with gastric cancer receiving first-line chemotherapy. Oncol Lett. (2020) 20:10. doi: 10.3892/ol.2020.11871 32774483 PMC7405604

[26] Sahni A Francis CW. Vascular endothelial growth factor binds to fibrinogen and fibrin and stimulates endothelial cell proliferation. Blood. (2000) 96:3772–8. doi: 10.1182/blood.V96.12.3772.h8003772_3772_3778 11090059

[27] Rickles F Patierno S Fernandez P. Tissue factor, thrombin, and cancer. Chest. (2003) 124:58S–68S. doi: 10.1378/chest.124.3_suppl.58S 12970125

[28] Levi M van der Poll T. Inflammation and coagulation. Crit Care Med. (2010) 38:S26–34. doi: 10.1097/CCM.0b013e3181c98d21 20083910

[29] Geranpayehvaghei M Dabirmanesh B Khaledi M Atabakhshi-Kashi M Gao C Taleb M. Cancer-associated-platelet-inspired nanomedicines for cancer therapy. Wiley Interdiscip Rev Nanomed Nanobiotechnol. (2021) 13:e1702. doi: 10.1002/wnan.1702 33538125

[30] Xu XR Yousef GM Ni H. Cancer and platelet crosstalk: opportunities and challenges for aspirin and other antiplatelet agents. Blood. (2018) 131:1777–89. doi: 10.1182/blood-2017-05-743187 29519806

[31] Demers M Ho-Tin-Noe B Schatzberg D Yang JJ Wagner DD. Increased efficacy of breast cancer chemotherapy in thrombocytopenic mice. Cancer Res. (2011) 71:1540–9. doi: 10.1158/0008-5472.CAN-10-2038 PMC3078642 21212409

[32] Bottsford-Miller J Choi HJ Dalton HJ Stone RL Cho MS Haemmerle M. Differential platelet levels affect response to taxane-based therapy in ovarian cancer. Clin Cancer Res. (2015) 21:602–10. doi: 10.1158/1078-0432.CCR-14-0870 PMC4315757 25473001

[33] Battinelli EM Markens BA Italiano JE Jr. Release of angiogenesis regulatory proteins from platelet alpha granules: modulation of physiologic and pathologic angiogenesis. Blood. (2011) 118:1359–69. doi: 10.1182/blood-2011-02-334524 PMC3152500 21680800

[34] Mitrugno A Sylman JL Ngo AT Pang J Sears RC Williams CD. Aspirin therapy reduces the ability of platelets to promote colon and pancreatic cancer cell proliferation: Implications for the oncoprotein c-MYC. Am J Physiol Cell Physiol. (2017) 312:C176–89. doi: 10.1152/ajpcell.00196.2016 PMC5336594 27903583

[35] Rothwell PM Wilson M Elwin C-E Norrving B Algra A Warlow CP. Long-term effect of aspirin on colorectal cancer incidence and mortality: 20-year follow-up of five randomized trials. Lancet. (2010) 376:1741–50. doi: 10.1016/S0140-6736(10)61543-7 20970847

[36] Spence AD Busby J Johnston BT Baron JA Hughes CM Coleman HG. Low-dose aspirin use does not increase survival in 2 independent population-based cohorts of patients with esophageal or gastric cancer. Gastroenterology. (2018) 154:849–860 e841. doi: 10.1053/j.gastro.2017.10.044 29122547

[37] Sun H Hu P Du J Wang X. Predictive value of inflammatory indexes on the chemotherapeutic response in patients with unresectable lung cancer: A retrospective study. Oncol Lett. (2018) 15:4017–25. doi: 10.3892/ol.2018.7781 PMC5796340 29467910

[38] Parkin DM Bray F Ferlay J Pisani P. Global cancer statistics, 2002. CA Cancer J Clin. (2005) 55:74–108. doi: 10.3322/canjclin.55.2.74 15761078

