# Supplementary material for: Platelet-lymphocyte ratio predicts chemotherapy response and prognosis in patients with gastric cancer undergoing radical resection
Source: Front Oncol. 2024 Mar 6;14:1279011. doi: 10.3389/fonc.2024.1279011 (PMC10951101; doi:10.3389/fonc.2024.1279011)

**Platelet-lymphocyte ratio predicts chemotherapy response and prognosis in patients with gastric cancer undergoing radical resection**

Qingnuo Zeng^1^, Danfang Wang^2^, Shilong Wang^1^, Zilong Bai^1^, Yuanhua Nie^1^, Longwen Xu^1^, Dongmin Chang^1, #^ and Dongmei Diao^1, #^

^1^Department of Oncology Surgery, First Affiliated Hospital of Xi’an Jiaotong University, Xi’an, China;

^2^Department of Xi'an Medical University, Xi’an, China;

^#^Corresponding Author: Dr. Dongmei Diao and and Prof. Dongmin Chang, Department of Surgical Oncology, First Affiliated Hospital Medical college Xi'an Jiaotong University, 277 West Yanta Road, Xi’an, Shaanxi 710061, P.R. China. Tel: 86-29-85324612, Fax: 86-29-85324612, E-mail: [**diaomei310@mail.xjtu.edu.cn**](mailto:diaomei310@mail.xjtu.edu.cn); sdmqqw@126.com.

**Supplementary Table 1. Univariate and multivariate analyses for disease-free survival of GC patients with low PLR (N=509).**

| Parameters | Univariate analysis | | | Multivariate analysis | | |
| --- | --- | --- | --- | --- | --- | --- |
|  | HR | 95%CI | P value | HR | 95%CI | P value |
| Gender | 1.151 | 0.8-1.655 | 0.449 |  |  |  |
| Age | 1.618 | 1.203-2.175 | 0.001 |  |  | 0.257 |
| FIB | 2.047 | 1.53-2.738 | <0.001 | 1.493 | 1.078-2.068 | 0.016 |
| SIRI | 1.292 | 0.97-1.722 | 0.08 |  |  |  |
| PNI | 0.537 | 0.389-0.741 | <0.001 |  |  | 0.352 |
| SII | 1.386 | 1.006-1.909 | 0.046 |  |  | 0.842 |
| NLR | 1.455 | 1.081-1.958 | 0.013 |  |  | 0.42 |
| LPR | 4.849 | 3.42-6.877 | <0.001 | 2.149 | 1.403-3.292 | <0.001 |
| Tumor size | 2.099 | 1.528-2.885 | <0.001 |  |  | 0.304 |
| Tumor location |  |  |  |  |  |  |
| proximal stomach | 1 |  |  |  |  |  |
| distal stomach | 0.604 | 0.431-0.845 | 0.003 |  |  | 0.525 |
| full stomach | 1.417 | 0.954-2.105 | 0.084 |  |  | 0.171 |
| Histology | 0.903 | 0.663-1.229 | 0.515 |  |  |  |
| TNM stage |  |  |  |  |  |  |
| I | 1 |  |  | 1 |  |  |
| II | 1.777 | 0.863-3.659 | 0.119 | 2.786 | 1.17-6.633 | 0.021 |
| III | 7.679 | 4.651-12.679 | <0.001 | 10.094 | 4.432-22.992 | <0.001 |
| Chemotherapy^1^ | 2.973 | 2.101-4.205 | <0.001 | 0.402 | 0.245-0.66 | <0.001 |

HR, hazard ratio; CI, confidence interval. The reference of parameters was female, age<60 years, FIB<3.585, PLR<163.8, SIRI<0.665, PNI<40.06, SII<456.3, NLR<2.08, LNR<0.085, tumor size<5 cm, undifferentiated and without adjuvant chemotherapy, respectively. ^1^In the univariate analysis considering only TNM stage II-III, the HR for adjuvant chemotherapy was 0.161 (95% CI: 0.113-0.228, P<0.001).

**Supplementary Table 2. Univariate and multivariate analyses for disease-free survival of GC patients with high PLR (N=221).**

| Parameters | Univariate analysis | | | Multivariate analysis | | |
| --- | --- | --- | --- | --- | --- | --- |
|  | HR | 95%CI | P value | HR | 95%CI | P value |
| Gender | 1.4 | 0.922-2.126 | 0.114 |  |  |  |
| Age | 1.634 | 1.105-2.416 | 0.014 | 1.601 | 1.044-2.457 | 0.031 |
| FIB | 1.12 | 0.768-1.633 | 0.557 |  |  |  |
| SIRI | 1.795 | 1.132-2.846 | 0.013 | 1.96 | 1.121-3.426 | 0.018 |
| PNI | 0.495 | 0.314-0.78 | 0.002 |  |  | 0.168 |
| SII | 1.247 | 0.751-2.068 | 0.393 |  |  |  |
| NLR | 1.133 | 0.737-1.743 | 0.569 |  |  |  |
| LNR | 3.569 | 2.292-5.559 | <0.001 | 3.314 | 2.125-5.168 | <0.001 |
| Tumor size | 1.314 | 0.867-1.99 | 0.198 |  |  |  |
| Tumor location |  |  |  |  |  |  |
| proximal stomach | 1 |  |  |  |  |  |
| distal stomach | 0.876 | 0.556-1.379 | 0.567 |  |  |  |
| full stomach | 1.571 | 0.88-2.803 | 0.127 |  |  |  |
| Histology | 0.812 | 0.525-1.256 | 0.349 |  |  |  |
| TNM stage |  |  |  |  |  |  |
| I | 1 |  |  |  |  |  |
| II | 2.219 | 0.885-5.564 | 0.089 |  |  | 0.214 |
| III | 4.224 | 1.954-9.133 | <0.001 |  |  | 0.634 |
| Chemotherapy^1^ | 1.525 | 0.991-2.347 | 0.055 |  |  | 0.055 |

The reference of parameters was female, age<60 years, FIB<3.585, PLR<163.8, SIRI<0.665, PNI<40.06, SII<456.3, NLR<2.08, LNR<0.085, tumor size<5 cm, undifferentiated and without adjuvant chemotherapy, respectively. ^1^In the univariate analysis considering only TNM stage II-III, the HR for adjuvant chemotherapy was 0.396 (95% CI: 0.254-0.616, P<0.001).

**Figure legends**

**Supplementary Figure 1.** ROC curves of inflammation factors. (a) ROC curve for PLR; (b) ROC curve for SIRI; (c) ROC curve for PNI; (d) ROC curve for SII; (e) ROC curve for NLR; (f) ROC curve for LNR; (g) ROC curve for FIB.


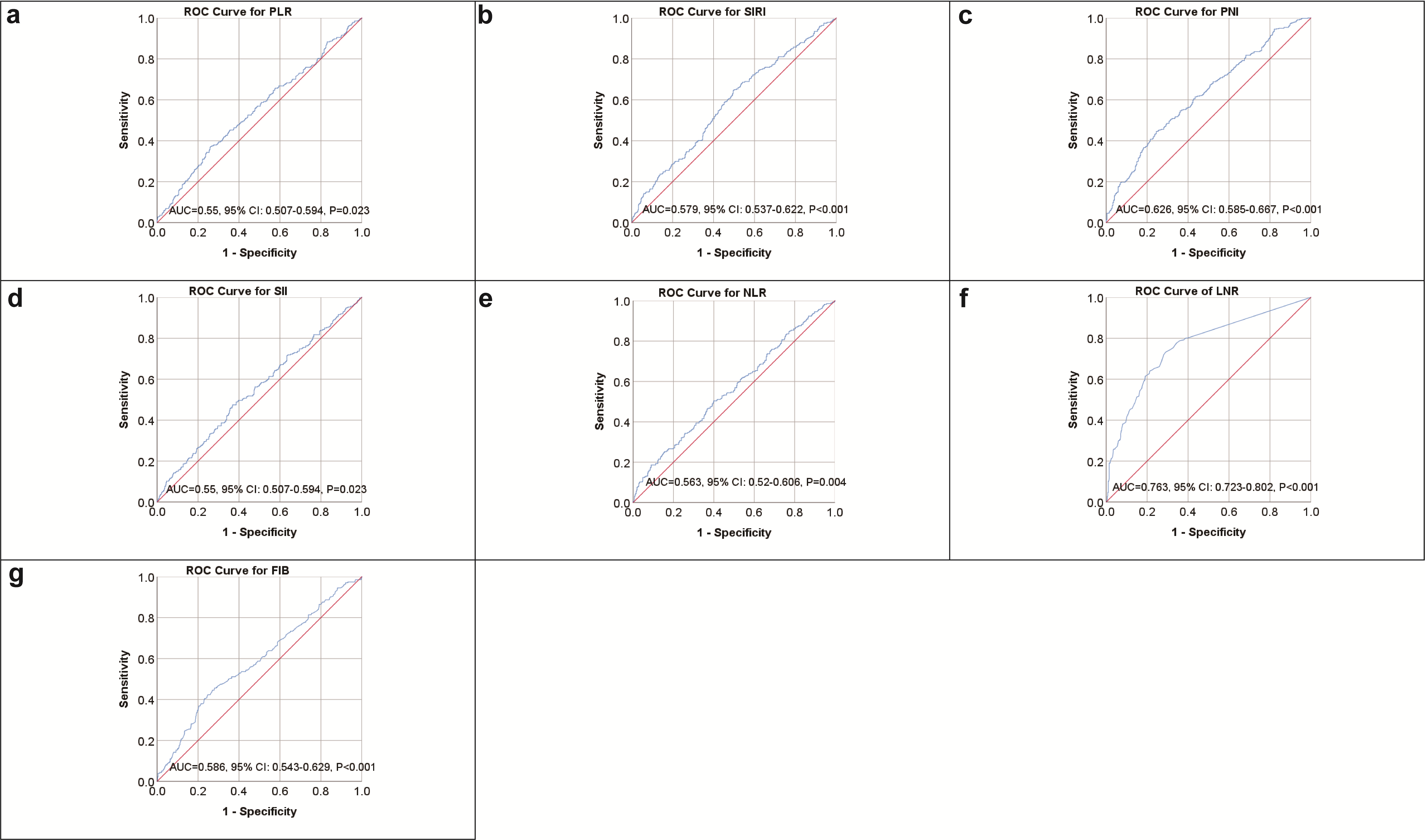


**Supplementary Figure 2.** Kaplan-Meier survival curves of patients with gastric cancer. (a) Survival curves of FIB for OS; (b) survival curves of FIB for DFS; (c) survival curves of SIRI for OS; (d) survival curves of SIRI for DFS; (e) survival curves of PNI for OS; (f) survival curves of PNI for DFS; (g) survival curves of SII for OS; (h) survival curves of SII for DF; (i) survival curves of NLR for OS; (j) survival curves of NLR for DFS.


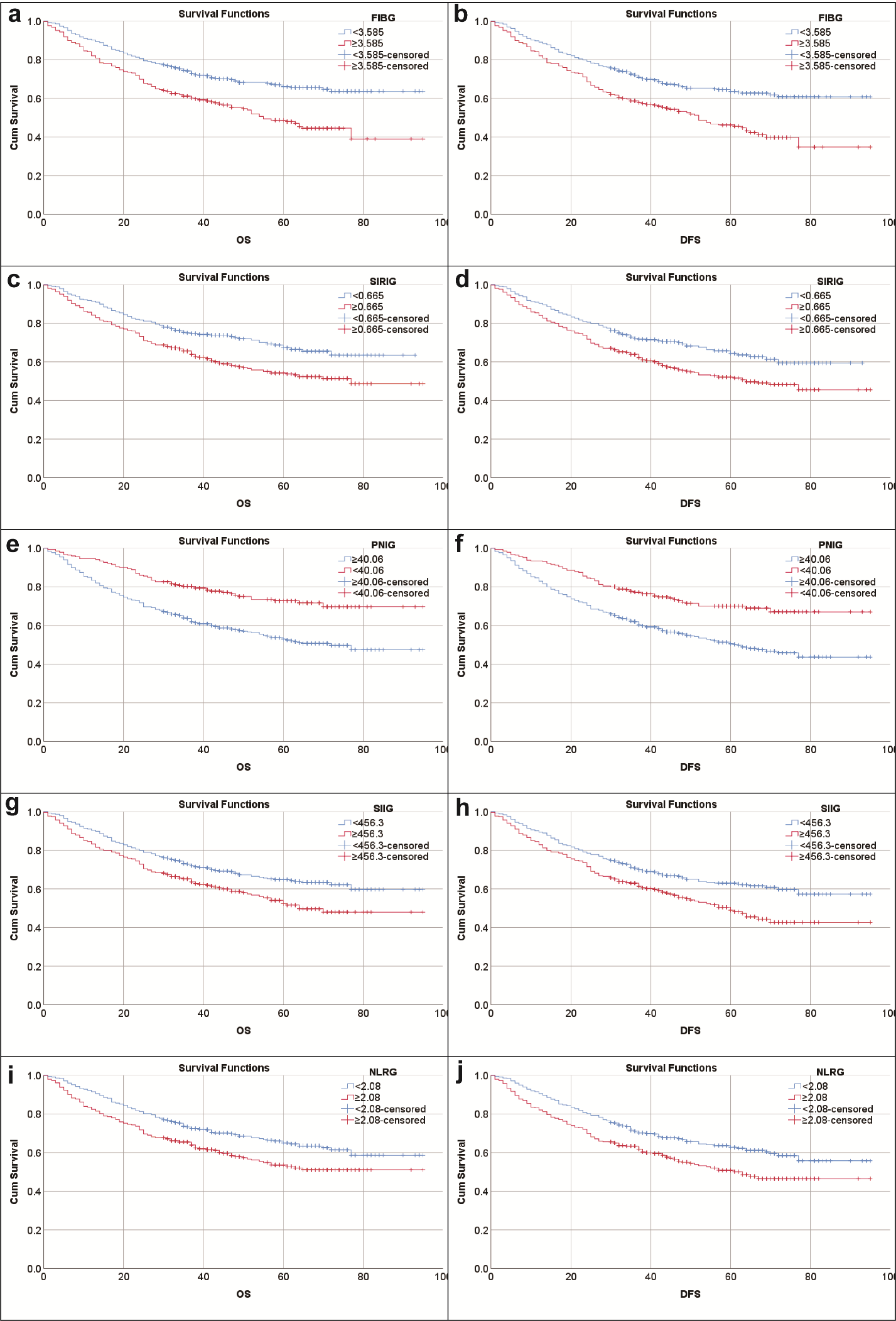


**Supplementary Figure 3.** Kaplan-Meier survival curves between PLR groups in patients receiving adjuvant chemotherapy. (a) Survival curve for OS of all patients receiving chemotherapy; (b) survival curve for DFS of all patients receiving chemotherapy; (c) survival curve for OS of stage II patients receiving chemotherapy; (d) survival curve for DFS of stage II patients receiving chemotherapy; (e) survival curve for OS of stage III patients receiving chemotherapy; (f) survival curve for DFS of stage III patients receiving chemotherapy.


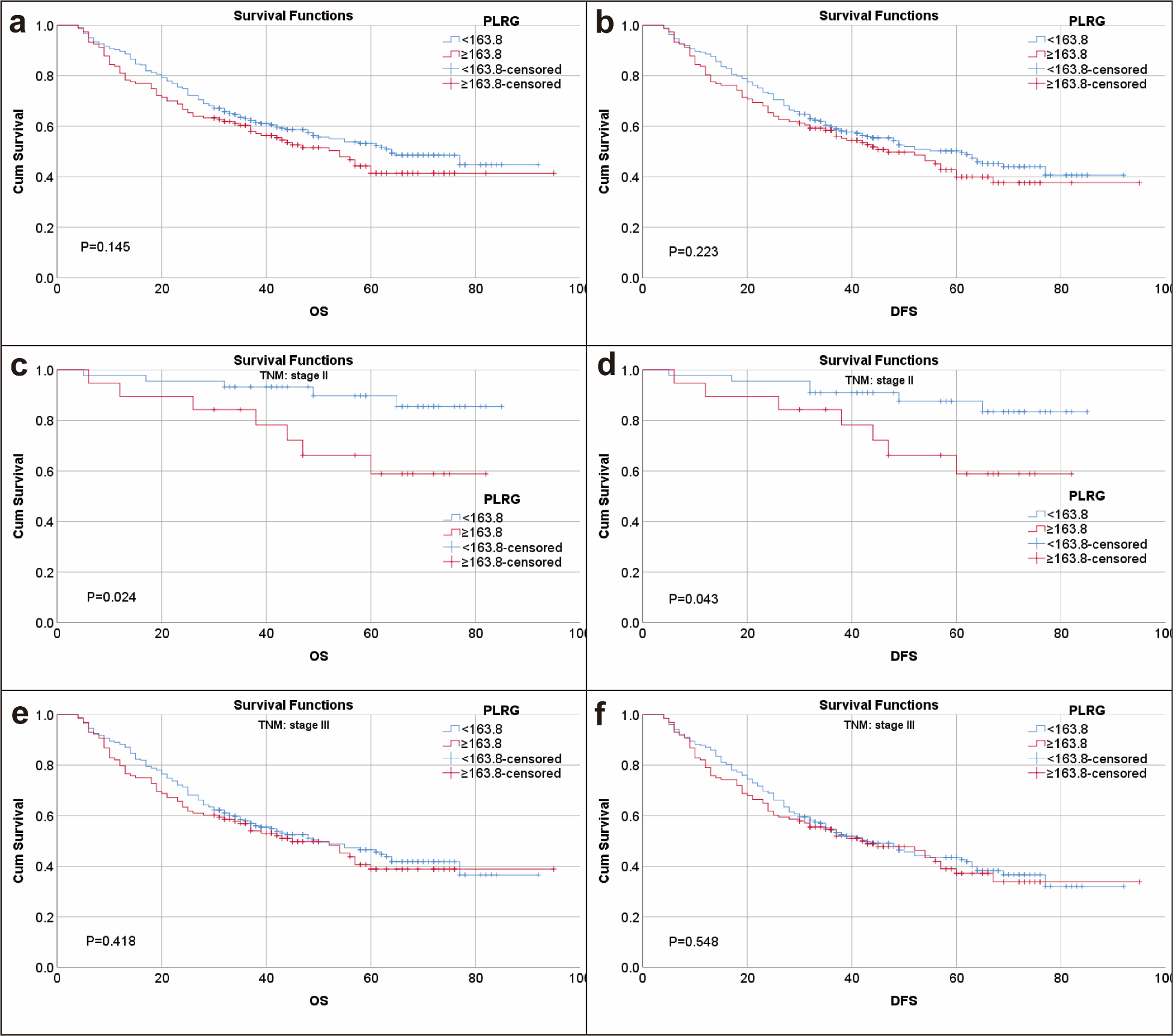

Supplement: Supplementary file 1 [file DataSheet_1.doc]
